# Observations on biotic parameters of Angora rabbit breed under controlled conditions in different housing systems

**DOI:** 10.14202/vetworld.2018.88-92

**Published:** 2018-01-30

**Authors:** Sajid Ur Rahman, Xichun Wang, Li Yu

**Affiliations:** Department of Clinical Veterinary medicine, College of Animal Science and Technology, Anhui Agricultural University, 130 West Changjiang Road, Hefei 230036, China

**Keywords:** body weight, heart rate, physiological parameters, respiratory rate, temperature

## Abstract

**Aim:**

The aim of the present study was to compare the body weight (BW) gain and physiological parameters such as temperature, respiratory rate (RR), and heart rate (HR) of Angora rabbit reared in different housing systems.

**Materials and Methods:**

A total of 30 angora rabbits (age 4-6 months), weight 1.5 kg in average were divided into three groups, i.e., (outdoor control [OC], indoor in cages [IC], and indoor open [IO]). All rabbits were reared for 10 weeks. Feed and water were given ad libitum. BW gain and physiological parameters such as temperature, RR, and HR were recorded.

**Results:**

All parameters showed some degree of variations. The BW differed significantly (p<0.05). The mean BW in kilogram (kg) of OC group was 1.59±0.03 obtained during the experimental period, while the BW of IC group shows a decrease of 1.43±0.05 and IO group it was 1.49±0.06 kg. The body temperature (BT) of the control group was 38.83±1.07°C, but IC and IO groups show increased in BT (39.10±0.78°C) and (39.33±1.24°C), indicated no significant difference among the groups (p=0.05). The RR in breaths/min of OC group recorded was 40.3±5.20, but the RR recorded for IC and IO groups was 41.2±7.29 and 39.3±6.30 breaths/min, respectively, showed less variation. The HR obtained in beat/min of OC group was 136.9±15.22, IC group (139.1±16.42) and IO group were (139.6±19.90 beat/min) showed less substantial variation.

**Conclusion:**

The present study clearly indicates that housing rabbits in cages and stress condition is a cause of poor welfare due to movement constraint, it will affect the body biotic parameters such as normal temperature, respiration as well as it can reduce the growth performance of animals significantly but housing system may not affect HR.

## Introduction

Rabbits are small mammals in the family Leporidae of the order Lagomorpha, found in several parts of the world [1]. There are eight different genera in the family classified as rabbits, including the European rabbit, cottontail rabbits, and the Amami rabbit. Animals kept at high temperature develop mechanisms to adapt to heat stress. In hot periods, rabbits have difficulty eliminating body heat because of their non-functional sweat glands [2]. At present, the most important topic in rabbit research is to improve the production taking into account the farmer requirements, animal welfare, and habitat. It is well known that rabbits are very sensitive to extreme environmental conditions, particularly temperature. The rabbits exposed to ambient temperature of 25°C for 12 h daily had lower weight gains than rabbits kept at 15°C. Environmental temperatures above 28°C cause heat-induced physiological stress. The critical air temperature for the quiet fasting rabbit is 27–28°C, which could be modified by humidity, hair coat, age, fatness, wind, and other factors, i.e., at air temperatures below 27°C a chemical regulation of rabbit BT comes into action [3,4]. This involves increased biological oxidation, resulting in increased heat production. When the air temperature rises beyond the upper limit of thermoneutrality range (32°C), physical regulation of BT is insured by the adjustment of blood flow to the skin and by the perspiration mechanisms. Vasomotor and cardio-respiratory mechanisms are also involved in addition to other physiological mechanisms. In general, chronic exposure to extremes of heat leads to decomposition of normal physiological and biological mechanisms with a consequent damage to many organs. Feed intake is reduced through high-temperature conditions; the growth is reduced consequently due to the decreased digestible energy intake [5].

The rising attention in obtaining meat from fewer intensive alternative housing systems has led to the implementation of “alternative rearing systems” in rabbit for these reasons, different “natural housing systems” have been studied [6-8]. The alternative rearing system needs breeds/populations characterized by a slow-growing rate, which can acclimatize to numerous environments, leading to satisfactory productive performance; in fact, the interaction among genotype, raising method and group size and density may disturb the animals’ welfare and production both positively and negatively. In recent years, studies carried out on a rabbit population with a slow development ratio presented their good productive performance, rusticity and capability to manage with dissimilar rearing systems, both organic and intensive [9]. Climatic heat stress has deleterious effects on exotic temperate breeds of rabbit’s more than indigenous tropical breeds.

The objectives of the conducted study were to examine the body weight gain (BWG) of the rabbit under controlled conditions and to determine the physiological parameters body temperature (BT), heart rate (HR), and respiratory rate (RR) of Angora rabbit under controlled conditions reared in different housing systems.

## Materials and Methods

### Ethics approval

The experimental protocol was approved by Anhui Agricultural University Animal Care and Institutional Animal Ethical Committee (Hefei, China).

### Study design

A total of 30 rabbits, aged between 4 and 6 months and weighing 1.5 kg in average were purchased from the local market and reared in Animal House for 10 weeks. The 30 rabbits (10 rabbits each group) were kept in different rearing condition divided into three groups. The first group is control group outdoor control (OC), and this group of rabbits was reared outdoor in open environment (not in cages) which has sufficiently available sunlight and natural air. They have freely available water and feed and also have enough space for walking, running, and jumping. The 2^nd^ group is an Indoor Group, housed in colony cages indoors (IC), and the 3^rd^ Indoor Group was reared openly without cages (IO). There is no sunlight provided to Group 2^nd^ and 3^rd^, (provide artificial light with the help of small light lamp a continuous 24-h light period was applied 8:00-24:00). The animals were fed complete feed and *ad libitum* alfalfa hay. Rabbits were individually identified by earmark, and their performance was controlled. Further details were reported by Xiccato *et al*. [10], including the growth performance obtained in this study. The animals observed 3 times daily. The health status of the rabbits was monitored daily to detect any clinical sign of diseases.

The floors roofs and walls of cages where the 2^nd^ group kept were made a wire net. The cage was equipped with a drinker and feeders (super pet gravity bin feeder) for the manual distribution of feed. During the experimental period, the rabbits were individually housed in universal galvanized wire batteries. The rabbits were individually weighed every week to investigate BWG or loss, and the data were recorded. To find alteration in rabbits physiological parameters their BT, HR, and RR was measured once in a week between 9:00 am and 11:00 am rather than the afternoon to avoid being inside the house during the severe period of heat stress.

The BW was measured using digital electric balance. The rectal temperature (RT) was recorded using a clinical thermometer inserted into the rectum for 2 min at a depth of 4 cm (Freudenberg medical Shenzhen, China). The HR was measured using a stethoscope (Freudenberg medical Shenzhen, China) by means of counting the heart beats per minute. The RR was recorded by counting the flank movements per minute using a hand counter. The behavior of the rabbits was video-recorded. During the night, the minimal light was used to avoid disturbing the activities of the rabbits. The following behaviors were analyzed as percentages of the observed time: Resting, jumping (crouched body, with abdomen in contact with the floor, or stretched body, with both fore and hind legs stretched beside the abdomen in contact with the floor), self-grooming, allogrooming, feeding, drinking, moving, running, standing still, biting, and sniffing [11,12].

### Statistical analysis

Cage data for growth performance were analyzed by ANOVA with the housing system as the main effect, using the SPSS statistics. Individual data for BW results, BT result, RR result, and HR were analyzed by ANOVA with the housing system as the main effect and the cage as the random effect. The experimental unit was the rabbit in case of BW and average weight gain; however, it was the pen in case of feed intake and feed conversion ratio. Differences among means with p<0.05 were accepted as statistically significant differences.

## Results

**Figure-1:**
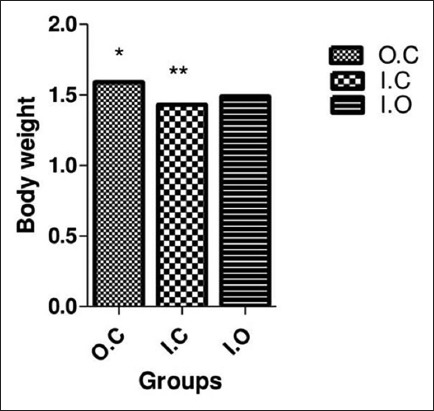
Group (G) body weight changes during 10 weeks. Data are expressed as mean±SD. In each row, the values with different stars indicate *significant difference among groups (p<0.05). OC=Outdoor control group, IC=Indoor cages, IO=Indoor open.

During the experiment, we found certain diseases in five rabbits in the IC group but only one rabbit in the IO group, while there is no any sign of disease found in the control group. The growth performance differed between groups. The BW of angora rabbits under control condition was differed significantly (p<0.05) after 10 weeks of experimental trials when compared the groups. Results presented in Figure-1 shows that BW was affected by housing system. The mean BW of control group during the experimental period was observed 1.59±0.03 kg compared to IC and IO groups which are 1.43±0.05 and 1.49±0.06 kg shows a significant difference (p<0.05) among groups. The poor growth performance of rabbits under management stress is probably influenced by biotic factors and housing system (Figure-1).

**Figure-2:**
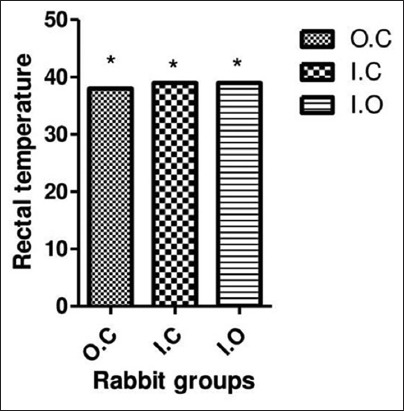
Group (G) overall body temperature changes throughout 10 weeks of the trial period. Data are expressed as mean±SD. The values with single star represent no significant difference (p=0.05) among groups. OC=Outdoor control group, IC=Indoor cages, IO=Indoor open.

Heat stress in rabbits induces a series of severe changes in their biological function and lead to the impairment in both production and reproduction. The results indicated a fluctuation in BT of animals during the research period, but there is no significant difference found among groups (Table-1). Figure-2 illustrates average RT for different groups. The data recorded for control group during the experimental trials were 38.83±1.07°C which is less variant as compared to IC and IO groups which showed average values of 39.10±0.78°C and 39.33±1.24°C, respectively. There is no significant difference found among groups (p=0.05); however, the variation in temperature is primarily related to biotic factors and rearing conditions and is not because of any ailment (Figure-2).

**Table 1:** BT (°C) and BW (kg) of Angora rabbits under control condition.

Week	BW				BT			
	
	OC	IC	IO	Mean±SD	OC	IC	IO	Mean±SD
1	1.50	1.65	1.40	1.51±0.12	40	40	41	40.3±1.03
2	1.50	1.65	1.40	1.51±0.12	38.89	39	39.44	39.11±0.52
3	1.45	1.60	1.55	1.53±0.07	39.05	39.11	39.44	39.01±0.20
4	1.40	1.60	1.55	1.51±0.10	38.78	38.89	38	38.85±0.11
5	1.35	1.55	1.55	1.48±0.11	39.05	39.44	39.11	39.20±0.37
6	1.45	1.60	1.50	1.51±0.07	39	39.44	39.44	39.30±0.46
7	1.45	1.55	1.55	1.51±0.05	38.33	39	38.39	38.68±0.66
8	1.45	1.60	1.55	1.53±0.07	37.87	38.97	38.78	38.48±0.10
9	1.45	1.55	1.45	1.48±0.05	39.16	38.88	39.45	39.96±0.50
10	1.35	1.55	1.40	1.43±0.10	38.33	38.38	39.44	38.72±1.12
Mean±SD	1.59±0.05	1.43±0.03	1.49±0.06	-	38.83±1.07	39.10±0.78	39.33±1.24	-

OC=Outdoor control, IC=Indoor cages, IO=Indoor open, SD=Standard deviation, BT=Body temperature, BW=Body weight

The data of Table-2 show some instability in RR rate during the trials era. Throughout the entire period, there was no any clinical sign of disease found related to respiration. The mean value of control group recorded was 40.3±5.20 stated as normal but both IC and IO group’s shows 41.2±7.29 and 39.3±6.30 breaths/min which indicated a significant difference (p<0.05) and decreased compared to control group. The results summarized in Table-2 also designated significant alteration during the research period in HR. To compare the entire three group’s performance, it shows substantial distinction because of the stress condition while rearing rabbits in cages. When compared with control group the HR beat/min shows irregularity, but there is no significant difference (p<0.05) found among groups as 136.9±15.22, 139.1±16.42, and 139.6±19.90 beat/min individually. It shows distinction because of the stress condition and poor welfare while rearing rabbits in cages in different housing system compared to the open and free environment (Table-2).

**Table 2:** RR (breath/min) and HR (beats/min) of angora rabbits under control condition.

Week	RR				HR			
	
	OC	IC	IO	Mean±SD	OC	IC	IO	Mean±SD
1	40	36	35	37.00±3.05	100	104	98	100.67±2.64
2	53	55	42	50.00±6.55	150	163	155	156.00±7.00
3	38	36	31	35.00±8.18	141	130	125	132.00±3.60
4	33	41	38	37.33±12.01	135	146	159	146.67±4.04
5	37	35	38	36.66±10.78	143	160	140	147.67±1.52
6	42	53	55	50.00±15.01	156	140	170	155.33±7.00
7	41	44	40	41.66±5.03	130	134	140	134.67±2.08
8	39	36	40	38.33±5.56	131	135	142	136.00±2.08
9	38	39	36	37.66±3.60	140	142	135	139.00±1.52
10	42	37	38	39.00±5.50	143	137	132	137.33±2.64
Mean±SD	40.3±5.20	41.2±7.29	39.3±6.30	-	136.9±15.22	139.1±16.42	139.6±19.90	-

OC=Outdoor control, IC=Indoor cages, IO=Indoor open, RR=Respiratory rate, HR=Heart rate, SD=Standard deviation

## Discussion

The best management system in rabbits is regarded as one of the most important economic traits in any breed development and improvement programs for intensive meat production. The growing rate differed significantly (p<0.05) between groups. The control group shows good performance and gaining weight quickly because of the free environment, but those rabbits which are kept in cages display a reduction in weight significantly (p<0.05). Dalle *et al*. [13] found that rabbits reared for meat production in cages decrease their production due to poor welfare and management system as well as lack of environmental interactions. The results in Figure-1 showed BW reduction is a consequence of fear and stress as well as housing system. Trocino *et al*. [12] and Verga *et al*. [14] also reported that rabbits in individual cages exhibited the maximum fear level and incomplete interactive patterns; the rabbits kept in collective cages showed the lowest fear levels and had the option of expressing a broader variety of behavior; and the rabbits in bicellular cages displayed an unpredictable design of fear in the tonic immobility and open-field tests. Probably, these rabbits were in a fewer stressful condition associated with animals in separate cages [15]. The poor growth performance of rabbits under management stress is probably influenced by biotic factors and housing system. Zeferino *et al*. [5] reported similar results that animal behaviors have become the major aspects of consideration in animal management during the experimental period because biotic factors mainly influence the body health of the animals.

Heat stress in rabbits makes a sequence of severe variations in their biological purpose which may lead to the loss of both production and reproduction. Bharathy *et al*. [16] observed that maximum and minimum temperature will be lower (p<0.05) in rabbits raised in platform shed than in conventional tile-roofed shed (p<0.01). The BT of angora rabbits fluctuates, but there is no any significant difference (p<0.05) found between all three groups. The results Table-1 indicated a fluctuation in BT of animals during the research period, but there is no substantial difference (p=0.05) found among groups. Siloto *et al*. [17] reported that mean daily temperature and relative humidity were higher in the natural temperature chamber than in the refrigerated one (23.6°C and 78.7% vs. 20.6°C, and 71.0%), respectively, which are close to values observed in the current study. In the natural temperature compartment, rabbits favored the wire net floor over the litter straw (77.9 vs. 22.1%, p<0.01), while in the refrigerated chamber they did not display any preference (45.9 vs. 54.1%, p=0.41) [18]. Adina *et al*. [19] found that ameliorating temperature effects especially in the afternoon could improve growth performance of rabbits in the tropics. Okab *et al*. [3] suggested that exposure of New Zealand rabbits to hot environmental conditions adversely affects physiological functions.

Table-2 presented some variability in RR breaths/min throughout the trials period shows a significant difference (p<0.05) among groups. During the entire period, there was no any clinical sign of disease seen related to respiration, but the variation may be because of stress and housing system which increase mean RR from 47 breaths/min to 55 breaths/min. The control group showed better adaptation with the environment because this group was kept in open environment outdoor with sufficient availability of fresh air as well as the animals were in less stressful condition compared with IC and IO group. Pavlova *et al*. [20] also found animals with prevailing right turnings in the open field displayed longer respiratory cycles and expirations than those with prevailing left turnings or housed in cages. It seems that the pattern of outdoor respiration can predict the inactive protective strategy of rabbits in negative emotional situations.

The results Table-2 also designated substantial changes in a heartbeat. Due to poor housing management and pressure, the Indoor Groups shows some variability during the current research, but there is no significant difference (p<0.05) detected. It shows that housing system has no effect on the heart of rabbits. Iyeghe *et al*. [21] reported pulse rates of 168 and 235 per minute they reported for New Zealand White is higher than was observed in this study. Fayez *et al*. [22] also detected a pulse rate of 137 cpm for Egyptian “Giza” rabbits under summer conditions are also higher than was observed in this study. Andrew *et al*. [23] reported that the biotic factors always affect the physical health of the animals.

These results indicate that housing system strictly affects growing performance BW as well as the physiological parameters BT and RR, but it may not affect the HR examined during current investigation. The rabbits reared in cages indoors increase loss of excess hair in cages, increase water intake, urination, panting, and lowered the activities. The variation in these parameters of rabbits was not the consequence of any disorder because rabbits were found fit and normal.

## Conclusion

Based on the data presented above and taking into account that housing rabbits in cages and stress condition is a cause of poor welfare due to movement constraint, it will affect the body biotic parameters such as normal temperature; respiration as well as it can reduce the growth performance of animals significantly. It was also concluded from the present study that housing system might not affect HR. Housing rabbits in IC or IO negatively affected certain traits, but the effects were more than reported in the previous studies.

## Authors’ Contributions

All authors contributed to the planning and doing research work as follows: SUR took charge of sample collection, doing, statistics, and writing the manuscript. WX and LY designed the study and gave valuable insights into the conduct of the study. All authors read and approved the final manuscript.
